# Integrated Characterization of ceRNA (circRNA/lncRNA/miRNA/mRNA) Regulatory Networks in a *Glaesserella parasuis*–Induced Mouse Meningitis Model

**DOI:** 10.3390/ani16172728

**Published:** 2026-09-02

**Authors:** Linrong Yang, Yingying Wu, Zhiyi Yan, Yanli Fei, Shulin Fu, Ling Guo, Zhe Chao

**Affiliations:** 1 Laboratory of Genetic Breeding, Reproduction and Precision Livestock Farming, School of Animal Science and Nutritional Engineering, Wuhan Polytechnic University, Wuhan 430023, China; ylr03250128@163.com (L.Y.); 19312420115@163.com (Y.W.); yanzhiyi2024@163.com (Z.Y.); fyl757302@163.com (Y.F.); shulinfu@whpu.edu.cn (S.F.); 2 Hubei Provincial Center of Technology Innovation for Domestic Animal Breeding, Wuhan 430023, China; 3 Hainan Key Laboratory for Tropical Animal Breeding and Disease Research, Institute of Animal Science and Veterinary Medicine, Hainan Academy of Agricultural Sciences, Haikou 571100, China

**Keywords:** *Glaesserella parasuis*, meningitis, circRNA, miRNA, lncRNA, ceRNA network

## Abstract

*Glaesserella parasuis* (*G. parasuis*, GPS) is a known causative agent of meningitis. A growing body of research indicates that non-coding RNAs (ncRNAs) are deeply involved in modulating inflammatory responses. In our study, we profiled the expression changes in circRNAs, lncRNAs, miRNAs, and mRNAs using a *G. parasuis*-induced mouse meningitis model and constructed the ceRNA networks by integrated analysis of the expression data. A total of 674 circRNAs, 376 lncRNAs, 57 miRNAs, and 373 mRNAs were differentially expressed. Functional GO and KEGG analysis showed enrichment of the source genes of differentially expressed (DE) circRNAs in protein binding, tight junction, and inflammatory pathways such as NF-kappaB, PI3K–Akt, and MAPK. The target genes of DEmiRNAs were enriched in phospholipase D, calcium, Rap1, and mTOR signaling pathways, whereas those of DElncRNAs were largely connected to tight junction, cytokine–cytokine receptor interaction, inflammatory response, and immune system processes. Subsequently, the DEcircRNAs–DEmiRNAs–DEmRNAs ceRNA network consisted of 177 DEcircRNAs, 20 DEmiRNAs, and 134 DEmRNAs, and the DElncRNAs–DEmiRNAs–DEmRNAs ceRNA network consisted of 152 DElncRNAs, 36 DEmiRNAs, and 177 DEmRNAs. Furthermore, the differential expression of randomly selected DEcircRNAs, DElncRNAs, DEmiRNAs, and DEmRNAs was confirmed by qRT-PCR, and the results were consistent with transcriptome sequencing. As far as we know, this study is the first integrative analysis for the ceRNA regulatory networks in *G. parasuis*-induced mouse meningitis, providing a theoretical basis for elucidating the pathogenic mechanisms of *G. parasuis* infection.

## Simple Summary

This study provides the first integrative analysis of ceRNA regulatory networks within a mouse model of meningitis triggered by *Glaesserella parasuis*. Unlike previous research focusing mainly on protein-coding genes, it highlights the crucial roles of non-coding RNAs in neuroinflammation. The findings offer new molecular insights that may guide biomarker discovery and therapeutic development for bacterial meningitis.

## 1. Introduction

*Glaesserella parasuis* (*G. parasuis*, GPS) is a gram-negative bacterial species that exhibits variable morphology and comprises over 15 distinct serotypes [1]. It is the causative agent of Glässer’s disease in pigs, which is characterized by fibrinous polyserositis, polyarthritis, and meningitis [2]. *G. parasuis* is widely distributed in the environment and can even be found colonizing in the upper respiratory tract of pigs without clinical signs [3]. When environmental conditions change or pigs are subjected to stress, *G. parasuis* can readily infect the upper respiratory tract, leading to disease in pigs [4]. *G. parasuis* has significantly impacted the global development of the swine industry, leading to substantial economic damage to pig production systems worldwide [5]. However, the pathogenicity of *G. parasuis* remains inadequately defined.

Non-coding RNAs (ncRNAs) are a diverse group of RNA molecules that lack protein-coding capacity but function as key modulators in various cellular activities [6]. Accumulating evidence indicates that non-coding RNAs, including circRNAs, lncRNAs, and miRNAs, play important regulatory roles in various pathological conditions, including infectious diseases, inflammatory disorders, and neurological diseases [6,7,8]. Notably, ncRNAs are deeply involved in modulating inflammatory responses, particularly in the context of diseases like meningitis, where inflammatory pathways are profoundly activated [7,8]. CircRNAs, a novel class of ncRNAs characterized by their covalently closed circular structure, have been implicated in regulating the immune response and inflammation [9]. Increasing research has demonstrated that circRNAs modulate the inflammatory response and blood–brain barrier (BBB) integrity by interacting with miRNAs or proteins, influencing gene expression during both the transcriptional and post-transcriptional levels [10]. For instance, circRNA_2858 has emerged as an important regulatory molecule in bacterial meningitis, where it modulates BBB disruption and inflammation via sponging miR-93-5p during bacterial meningitis [11]. Similarly, circRNA_PTPN4 has been reported to enhance FOXO3 expression by functioning as a molecular sponge for miR-301a-3p, which subsequently increases ZO-1 expression and restores the BBB integrity in mice with uremic encephalopathy [12]. Furthermore, long non-coding RNAs (lncRNAs) are recognized as being able to participate in human meningitis and are identified as biomarkers [13,14]. LncRNA NEAT1, for example, impaired the integrity and increased the permeability of BBB by interacting with miR-135a, thus contributing to the inflammatory cascade seen in bacterial meningitis [15]. LncRNA IMAT1 enhances meningioma cell invasion by serving as a competitive binder of hsa-miR22-3p, which leads to the inactivation of the KLF4/hsa-miR22-3p/Snai1 pathway, and IMAT1 may represent a promising diagnostic indicator and therapeutic candidate for meningiomas [16]. MiRNAs, small non-coding RNAs that post-transcriptionally regulate the expression of target genes, are also critical players in meningitis [17,18]. miR-155 and miR-146a collaboratively regulate bacteria-induced neuroinflammatory responses through negative feedback regulation involving TLR-mediated NF-κB and EGFR–NF-κB signaling pathways [19]. In addition, increasing evidence suggests that non-coding RNAs are involved in coagulation-related processes by regulating the expression of coagulation factors, platelet activation, and inflammatory–coagulation crosstalk [20].

However, the regulatory relationships and interaction networks of circRNAs, lncRNAs, and miRNAs have not been fully elucidated, especially in the pathogenic processes associated with meningitis caused by *G. parasuis*.

The concept of competing endogenous RNA (ceRNA) networks has emerged as a key mechanism of ncRNA regulation, playing a critical role in the regulation of neurological disorders [21,22]. In this model, different RNA molecules—primarily circRNAs, lncRNAs, and miRNAs—interact with one another through miRNA response elements (MREs), thereby inhibiting the silencing of miRNA target mRNAs [21]. The ceRNA network is thought to regulate the inflammation process by balancing miRNA availability. In addition to neurological disorders, ceRNA networks have also been implicated in inflammatory responses across various diseases, including atherosclerosis [23], cancers [24,25], hypertrophic cardiomyopathy [26], and obesity [27]. Another study reported that inflammatory mediators and signaling pathways are effectively modulated by ceRNA regulatory networks in atherosclerosis [23]. Thus, through post-transcriptional regulation and ceRNA-mediated interactions, these molecules can influence immune responses, inflammatory signaling, and host–pathogen interactions [21,22,23]. However, the role of ceRNA networks in meningitis remains poorly understood, and the potential involvement of circRNAs and ceRNA networks in this context has yet to be explored.

In this study, we employed our previously established mouse model of *G. parasuis*-induced meningitis [28] to investigate the ceRNA regulatory network involved in the inflammatory response following infection. Combined with whole-genome sequencing and quantitative reverse-transcribed PCR, we profiled the expression changes in circRNAs, lncRNAs, miRNAs, and mRNAs during brain inflammation. Based on these expression profiles, we constructed ceRNA networks to explore the interaction relationship and the roles of circRNAs, lncRNAs, miRNAs, and mRNAs in *G. parasuis*-induced meningitis, aiming to address the knowledge gap in this area.

## 2. Materials and Methods

### 2.1. Experimental Animals and Bacteria

Sixty female Kunming mice (six weeks old) were obtained from Hubei Yi Zhicheng Biotechnology Co., Ltd. (Wuhan, China). Upon arrival at the animal facility, all animals were acclimatized for 7 days before the experiments. Mice were housed under specific pathogen-free conditions with free access to food and water. The *G. parasuis* SH0165 strain originated from the State Key Laboratory of Agricultural Microbiology at Huazhong Agricultural University (Wuhan, China). SH0165 is a highly virulent serovar 5 strain that has been extensively used in studies of *G. parasuis* pathogenesis. To ensure consistency with our previous studies, SH0165 was selected for establishing the infection model. The bacterial strain was cultured in tryptic soy broth (Becton, Dickinson and Company, Franklin Lakes, NJ, USA) containing 10 μg/mL nicotinamide adenine dinucleotide (Beyotime Biotechnology, Wuhan, China) and 10% fetal bovine serum (Zhejiang Tianhang Biotechnology Co., Ltd., Huzhou, China). Animal experiments were approved by the Animal Care and Use Committee of Wuhan Polytechnic University, Hubei Province, China (Approval No. WPU202306001). All experimental procedures were performed in accordance with institutional guidelines and regulations and were reported following the ARRIVE (Animal Research: Reporting of In Vivo Experiments) guidelines. 

### 2.2. Experimental Design

The mouse infection model was established as previously described by our group [28], with the same bacterial strain, infection route, and experimental conditions. A total of 60 female Kunming mice were randomly allocated into six independent groups (n = 10 per group), including control and infected groups at 24, 48, and 72 h post-infection. At each time point, an independent cohort of 10 control mice and 10 infected mice was euthanized for sample collection and subsequent analyses. The *G. parasuis* group received an intraperitoneal injection of 2 × 10^9^ CFU (CFU, colony-forming unit) of *G. parasuis* suspended in 0.2 mL saline, while the control group received equivalent saline. Disease status was monitored twice daily after *G. parasuis* infection at 24, 48, and 72 h post-infection, including activity, posture, neurological symptoms, food intake, and general health status. Mice were deeply anesthetized with sodium pentobarbital (50 mg/kg, intraperitoneally) and subsequently euthanized by cervical dislocation. Following dissection, the mouse brain samples were isolated. For molecular analyses, six tissue samples were collected from anatomically distributed sampling sites across the whole brain to obtain representative tissue from different areas, and these samples were pooled and immediately snap-frozen in liquid nitrogen and stored at −80 °C until RNA extraction. The sample size was determined based on our previous experience with this established mouse model.

### 2.3. Hematoxylin and Eosin (H&E) Staining

For histological analysis, tissue sections were collected from the same anatomically defined location within the cerebrum of each mouse at 24, 48, and 72 h post-infection, using a consistent sampling and sectioning procedure to ensure comparability among experimental groups and time points. The tissues were fixed in 4% paraformaldehyde for subsequent histological processing and H&E staining. After that, brain tissues were paraffin-embedded, sectioned, and stained with hematoxylin and eosin (H&E) using standard histological procedures. Histopathological evaluation was performed by a veterinary pathologist experienced in histological assessment and blinded to the experimental group allocation.

### 2.4. Total RNA Extraction

Total RNA was extracted from mouse brain tissues using the Trizol reagent kit (Takara, Beijing, China), according to the manufacturer’s instructions. The concentration and purity of RNA were determined using a Merinton SMA5000 spectrophotometer (Merinton, Beijing, China), and RNA integrity was evaluated before subsequent RNA sequencing and qRT-PCR analyses.

### 2.5. Transcriptome Sequencing and Data Analysis

Qualified total-RNA samples were used for library construction. For mRNA and lncRNA sequencing, rRNA-depleted RNA libraries were constructed using a strand-specific RNA sequencing protocol. For circRNA sequencing, ribosomal RNA depletion followed by linear RNA removal was performed to enrich circular RNA molecules before library construction. For miRNA sequencing, small RNA libraries were generated by enriching small RNA fragments, followed by adapter ligation, reverse transcription, and PCR amplification. All libraries were subjected to quality assessment and sequenced on Illumina NovaSeq 6000 platform by Genedenovo Biotechnology Co., Ltd. (Guangzhou, China). Clean reads were aligned to the ribosomal RNA database using Bowtie2 (v2.5.4, http://bowtie-bio.sourceforge.net/bowtie2, accessed on 21 March 2025) and then aligned to the reference genome (Ensembl_release110) using HISAT2 (v2.2.1, https://daehwankimlab.github.io/hisat2/, accessed on 30 March 2025). Circular RNAs were identified based on back-splicing junction detection, while lncRNAs were annotated according to transcript characteristics and coding potential. Small RNA reads were processed for miRNA identification and annotation based on known miRNA databases. Differential expression analysis was conducted using edgeR (v4.2.2, https://bioconductor.org/packages/release/bioc/html/edgeR.html, accessed on 6 April 2025), which applies a negative binomial model for RNA-seq count data. Multiple testing correction was performed using the false discovery rate (FDR). CircRNAs, lncRNAs, and mRNAs with FDR < 0.05 and |log_2_FC| > 1, and miRNAs with *p* < 0.05 and |log_2_FC| > 1, were considered significantly differentially expressed.

### 2.6. Prediction of circRNAs

The clean reads were aligned to the reference genome with high mapping quality and then submitted to CIRIquant (v1.1.3, https://github.com/bioinfo-biols/CIRIquant, accessed on 10 April 2025) for circRNAs identification. Reliable circRNAs were obtained after filtering based on reads alignment statistics, annotation categories, and genomic distribution.

### 2.7. Functional Enrichment Analysis

GO and KEGG pathway enrichment analysis were performed for the source genes of differentially expressed circRNAs (DEcircRNAs), the target genes of DEmiRNAs and DElncRNAs in *G. parasuis*-infected mouse brain. The analysis of the target genes of lncRNAs was based on cis-, antisense-, and trans-acting predictions. GO and KEGG pathway enrichment analyses were performed based on the over-representation analysis (ORA) using the hypergeometric test. The significance of enriched terms or pathways was evaluated by calculating the probability of observing the overlap between the input gene set and the annotated gene sets under a hypergeometric distribution. *p* values were adjusted for multiple testing, and adjusted *p* values < 0.05 were considered statistically significant.

### 2.8. Construction of Interaction Networks Among the Molecules Associated with Inflammatory Pathways

Inflammatory pathways, including NF-kappa B, JAK–STAT, MAPK, and PI3K–Akt signaling pathways, were selected based on KEGG pathway enrichment analysis of the source genes of DEcircRNAs. Subsequently, DEmRNAs involved in these inflammation-related pathways were identified according to the significant difference. The corresponding DEmiRNAs targeting these DEmRNAs and the DEcircRNAs interacting with these DEmiRNAs were further selected based on the predicted regulatory relationships. Separate interaction networks (DEcircRNAs–DEmiRNAs–DEmRNAs) were constructed and visualized using Cytoscape software (v3.10.1, http://www.cytoscape.org/).

### 2.9. ceRNA Regulatory Networks Construction

ceRNA regulatory networks in *G. parasuis*-infected mouse brain were constructed based on the negatively correlated expression patterns between DEmiRNAs and DEcircRNAs/DElncRNAs/DEmRNAs. The expression correlation was assessed using the Spearman Rank correlation coefficient (SCC), and pairs with coefficients less than −0.7 were considered negatively co-expressed. The co-expression competing triplets were then assembled into DEcircRNAs–DEmiRNAs–DEmRNAs and DElncRNAs–DEmiRNAs–DEmRNAs networks, which were visualized using Cytoscape (v3.10.1).

### 2.10. Validation Using Quantitative Reverse Transcription PCR (qRT-PCR)

qRT-PCR was performed on the mouse brain tissues infected with *G. parasuis* for 72 h to validate the expression of inflammatory cytokines, randomly selected DEcircRNAs, DElncRNAs, DEmiRNAs, and DEmRNAs. U6 served as the endogenous control for miRNAs, while GAPDH did for circRNAs, lncRNAs, and mRNAs. The primer sequences are shown in Table 1. Statistical analyses were performed using GraphPad Prism software 8.3.0 (GraphPad Software, San Diego, CA, USA). Comparisons between two groups were performed using Student’s *t*-test. Data are presented as mean ± SD (n = 3), and *p* < 0.05 was considered statistically significant.

## 3. Results

### 3.1. G. parasuis Infection Caused Meningitis Symptoms in Mice

**Figure 1:**
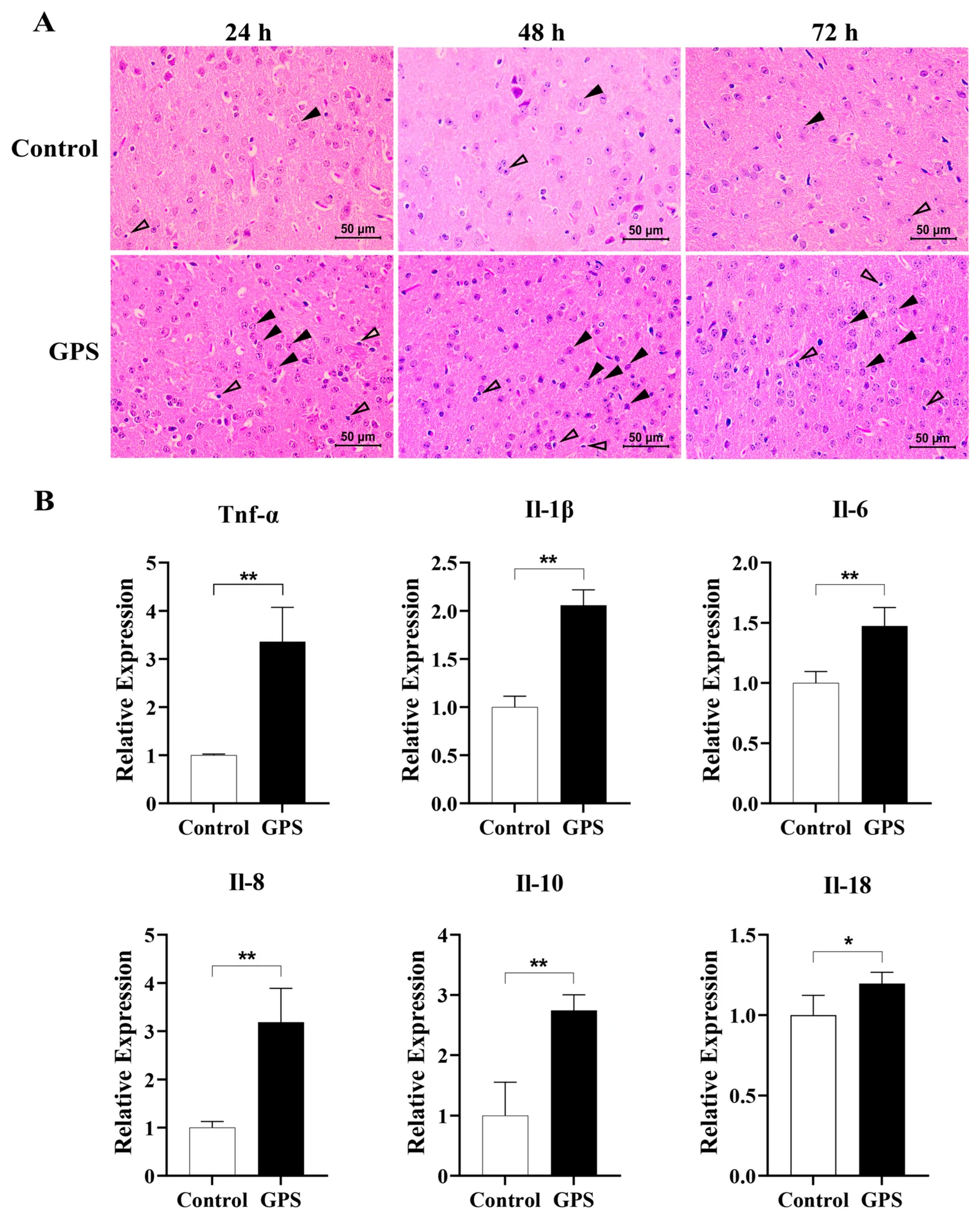
Hematoxylin and eosin (H&E) staining and relative expression of inflammatory cytokines in mouse brain following *G. parasuis* infection. (**A**) Representative H&E-stained sections of mouse brain tissues at 24, 48, and 72 h following *G. parasuis* infection (magnification: 10 × 40). Black solid arrows indicate neutrophils, and black hollow arrows indicate monocytes. (**B**) The relative expression levels of inflammatory cytokines (Tnf-α, Il-1β, Il-6, Il-8, Il-10, and Il-18) in the mouse brain at 72 h post *G. parasuis* infection. Data are presented as mean ± SD (n = 3). Statistical significance was determined using Student’s *t*-test. * *p* < 0.05 and ** *p* < 0.01 versus the control group.

Brain tissues were collected from *G. parasuis*-infected mice at 24, 48, and 72 h after infection, and pathological sections were prepared and stained with H&E. Qualitative morphological observations showed relatively intact cerebral architecture without obvious inflammatory cell aggregates in the control mice. In contrast, an apparent accumulation of small, hyperchromatic inflammatory cells was observed in the cerebral tissues of *G. parasuis*-infected mice at 24, 48, and 72 h, accompanied by variable tissue rarefaction and vacuolation. Black arrowheads indicate representative areas of inflammatory cell accumulation. These findings qualitatively suggest that *G. parasuis* infection induced an inflammatory response in the mouse brain (Figure 1A). In addition, the qRT-PCR assay was employed to examine changes in the expression of inflammatory cytokines. Compared to the control group, significant increases in Tnf-α, Il-1β, Il-6, Il-8, Il-10, and Il-18 expression were detected in the *G. parasuis*-infected group (72 h) (*p* < 0.05) (Figure 1B), indicating that *G. parasuis* infection induced the inflammatory response in the mouse brain.

### 3.2. Identification of DEcircRNAs, DElncRNAs, DEmiRNAs, and DEmRNAs in G. Parasuis-Infected Mouse Brain

**Figure 2:**
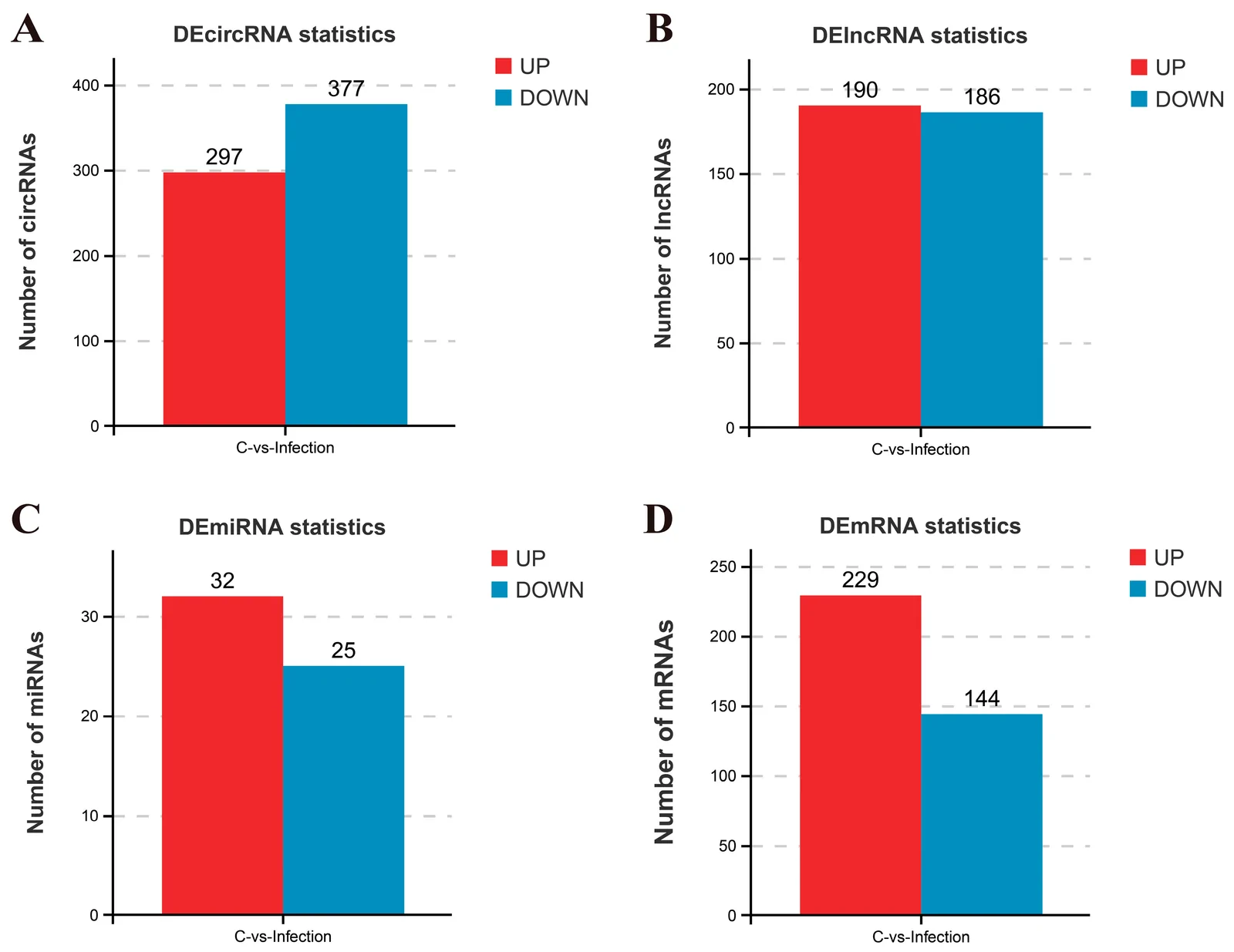
The numbers of circRNAs, lncRNAs, miRNAs, and mRNAs showing differential expression in *G. parasuis*-infected mouse brain. (**A**) The numbers of DEcircRNAs in *G. parasuis*-infected mouse brain. (**B**) The quantities of DElncRNAs in mouse brain after *G. parasuis* infection. (**C**) The numbers of DEmiRNAs in *G. parasuis*-infected mouse brain. (**D**) The quantities of DEmRNAs in *G. parasuis*-infected mouse brain. CircRNAs, lncRNAs, and mRNAs: FDR < 0.05 and |log2FC| > 1 as significant difference; miRNAs: *p* < 0.05 and |log2FC| > 1 as significant difference.

Transcriptome sequencing was performed on the brain tissues of mice infected with *G. parasuis* for 72 h to profile the transcriptional differences. The 72 h post-infection time point was selected based on the progression of infection-associated inflammatory responses observed in this model and our previous experience with this infection system. Compared to the control group, the *G. parasuis*-infected group showed an overall count of 674 DEcircRNAs (297 upregulated and 377 downregulated circRNAs), 376 DElncRNAs (190 upregulated and 186 downregulated), 57 DEmiRNAs (32 upregulated and 25 downregulated), and 373 DEmRNAs (229 upregulated and 144 downregulated) (*p* < 0.05, Figure 2A–D).

### 3.3. Identification and Characterization of circRNAs in Mouse Brain Following G. Parasuis Infection

**Figure 3:**
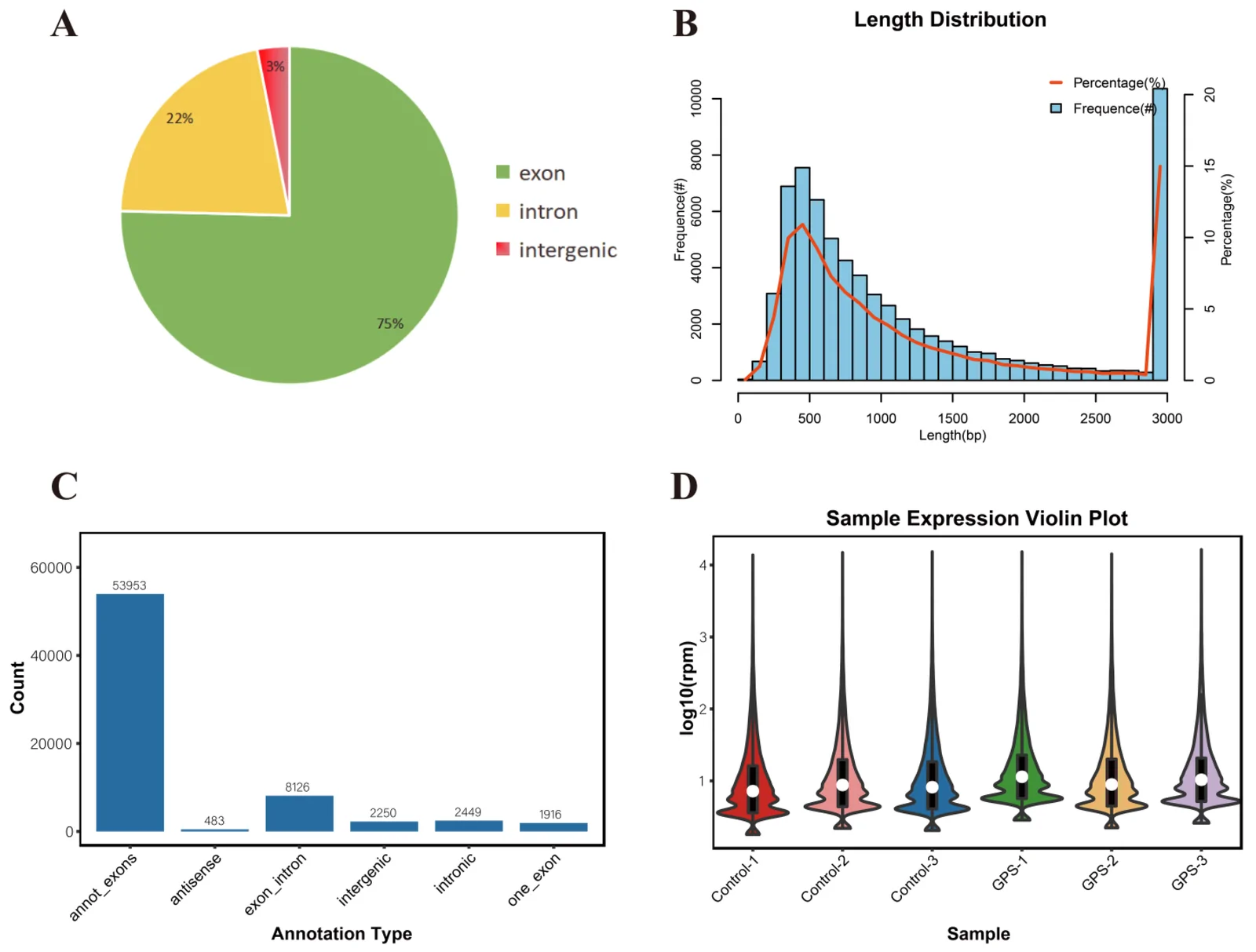
Identification and characteristics of circRNAs in mouse brain following *G. parasuis* infection. (**A**) Reads align region statistics in mouse brain after *G. parasuis* infection. (**B**) Length distribution of circRNAs in *G. parasuis*-infected mouse brain. (**C**) Annotation types of circRNAs in *G. parasuis*-infected mouse brain. Annot_exons: circRNAs originate from annotated exons. Antisense: circRNAs originate from the antisense strand. Exon_intron: circRNAs originate from both exonic and intronic regions. Intergenic: circRNAs originate from intergenic regions. Intronic: circRNAs originate from introns. One_exon: circRNAs are composed of a single exon. (**D**) Sample violin plot of circRNAs in the mouse brain between the control and *G. parasuis* groups.

Based on the alignment results of all reads mapped to the genome (Total_Mapped reads), the distribution of reads across the reference genome (Ensembl_release110) was calculated. The aligned regions were classified into exonic regions (exon), intronic regions (intron), and intergenic regions (intergenic), with the majority of the reads mapping to the exonic regions (Figure 3A). Following reads alignment to the reference genome, circRNAs were identified by CIRIquant (v1.1.3, https://github.com/bioinfo-biols/CIRIquant, accessed on 10 April 2025). Subsequently, statistics were conducted on circRNA types and length distribution. Results showed that circRNAs with the lengths of 300–500 nt and approximately 3000 nt were the most abundant (Figure 3B). In addition, the majority of circRNAs were derived from the annotated exons, followed by those spanning both exonic and intronic regions of genes (Figure 3C). A sample expression violin plot was generated to visualize the data distribution and probability density, thereby illustrating the overall distribution patterns. As depicted in Figure 3D, the expression abundance and density of circRNAs in the control group and *G. parasuis*-infected group were well represented.

### 3.4. Functional Enrichment Analysis for the Source Genes of DEcircRNAs and the Target Genes of DEmiRNAs in G. Parasuis-Infected Mouse Brain

**Figure 4:**
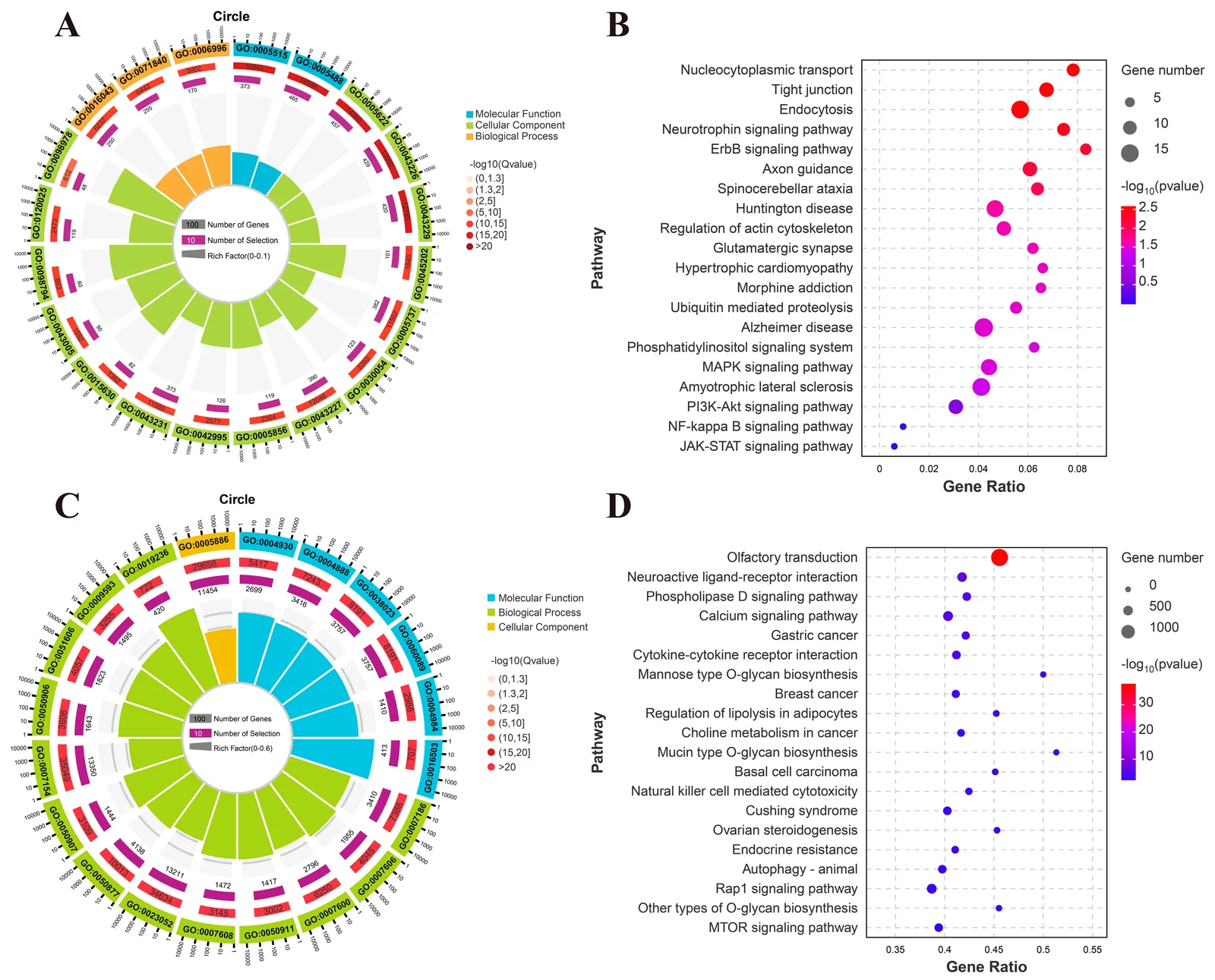
Functional enrichment analysis of the source genes of DEcircRNAs and the target genes of DEmiRNAs in *G. parasuis*-infected mouse brain. (**A**) Circle diagram of GO analysis for the source genes of DEcircRNAs. (**B**) KEGG analysis for the source genes of DEcircRNAs. (**C**) GO enrichment circle diagram of the target genes of DEmiRNAs. (**D**) KEGG analysis of the target genes of DEmiRNAs.

Following the identification of DEcircRNAs through differential expression analysis, functional enrichment analysis of the source genes of DEcircRNAs was carried out to explore the potential biological roles of DEcircRNAs in mouse brain after *G. parasuis* infection. GO functional profiling revealed that the source genes of DEcircRNAs were predominantly enriched in protein binding (GO:0005515), cell junction (GO:0030054), cytoskeleton (GO:0005856), and protein localization (GO:0008104) (FDR ≤ 0.05, Figure 4A). Meanwhile, KEGG pathway analysis indicated that the source genes of DEcircRNAs were primarily involved in tight junction, endocytosis, ErbB, neurotrophins, MAPK, PI3K–Akt, NF-kappa B, and JAK–STAT signaling pathways (FDR ≤ 0.05, Figure 4B). On the other hand, the most significantly enriched GO terms of the DEmiRNA targets were G protein-coupled receptor activity (GO:0004930), transmembrane signaling receptor activity (GO:0004888), sensory perception of chemical stimulus (GO:0007606), and detection of stimulus (GO:0051606) (FDR ≤ 0.05, Figure 4C). Meanwhile, further KEGG pathway analysis revealed that the target genes of DEmiRNAs were mainly associated with olfactory transduction, phospholipase D, calcium, Rap1, and mTOR signaling pathways (FDR ≤ 0.05, Figure 4D). These findings suggested that DEcircRNAs and DEmiRNAs regulated genes involved in signal transduction, cellular communication, and inflammatory response. Notably, the enrichment of signaling pathways such as NF-kappa B, JAK–STAT, and MAPK implied that these differential ncRNAs played critical roles in mediating inflammatory processes in mouse brain during *G. parasuis* infection.

### 3.5. GO and KEGG Enrichment Analysis for the Target Genes of lncRNAs in G. Parasuis-Infected Mouse Brain

**Figure 5:**
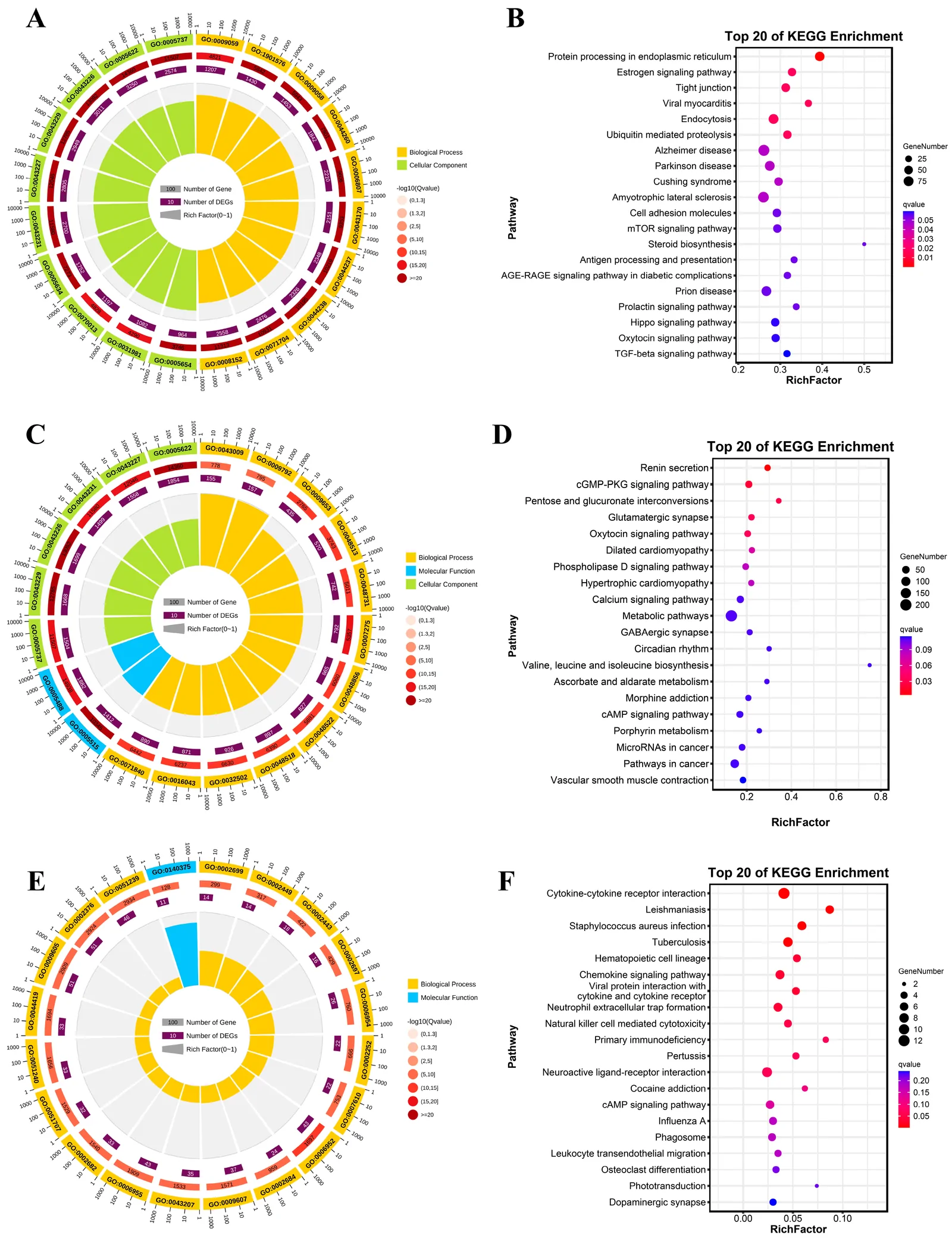
GO and KEGG enrichment analysis of the target genes of lncRNAs in mouse brain following *G. parasuis* infection. (**A**,**B**) GO and KEGG analysis of the target genes of lncRNAs by cis-acting prediction. (**C**,**D**) GO and KEGG analysis of the target genes of lncRNAs through antisense-acting prediction. (**E**,**F**) GO and KEGG analysis of the target genes of lncRNAs by trans-acting prediction.

To further elucidate the potential functional regulation of lncRNAs, GO and KEGG analysis were performed on the target genes of lncRNAs in *G. parasuis*-infected mouse brain, including target genes predicted by cis-acting, antisense-acting, and trans-acting. Firstly, GO enrichment analysis for the cis-acting predicted targets demonstrated that the target genes of lncRNAs were primarily connected to the biosynthetic process (GO:0009058), cellular macromolecule metabolic process (GO:0044260), organic substance metabolic process (GO:0071704), intracellular anatomical structure (GO:0005622), and nucleus (GO:0005634) (FDR ≤ 0.05, Figure 5A). Correspondingly, KEGG analysis demonstrated that the target genes of lncRNAs were predominantly concentrated in the endocytosis, estrogen signaling pathway, tight junction, ubiquitin-mediated proteolysis, and mTOR signaling pathway (FDR ≤ 0.05, Figure 5B). Secondly, GO analysis using the target genes predicted by antisense-acting indicated that the target genes of lncRNAs were mainly enriched in protein binding (GO:0005515), cellular component organization or biogenesis (GO:0071840), positive regulation of the cellular process (GO:0048522), and animal organ development (GO:0048513) (FDR ≤ 0.05, Figure 5C), and the corresponding KEGG pathways were enriched in the cGMP–PKG signaling pathway, glutamatergic synapse, phospholipase D signaling pathway, calcium signaling pathway, metabolic pathways, and cAMP signaling pathway (FDR ≤ 0.05, Figure 5D). In addition, GO enrichment analysis for the trans-acting predicted targets indicated that the target genes of lncRNAs were largely associated with immune receptor activity (GO:0140375), the immune system process (GO:0002376), the response to external stimuli (GO:0009605), the defense response (GO:0006952), and the inflammatory response (GO:0006954) (FDR ≤ 0.05, Figure 5E). Simultaneously, KEGG analysis demonstrated that these target genes had significant enrichment in cytokine–cytokine receptor interaction, viral protein interaction with cytokines and cytokine receptors, neuroactive ligand–receptor interaction, natural killer cell-mediated cytotoxicity, primary immunodeficiency, and the cAMP signaling pathway (FDR ≤ 0.05, Figure 5F). Collectively, these findings imply that the identified lncRNAs may be involved in key biological processes such as neurological cell communication, tight junction integrity, and immune response. This suggests their potential pivotal roles in mediating host–pathogen interactions and immune modulation in mouse brain during *G. parasuis* infection. However, these proposed functions require further experimental validation in future studies. 

### 3.6. The Regulatory Networks Among Molecular Signatures Associated with the Inflammatory Signaling Pathways in G. Parasuis-Infected Mouse Brain

**Figure 6:**
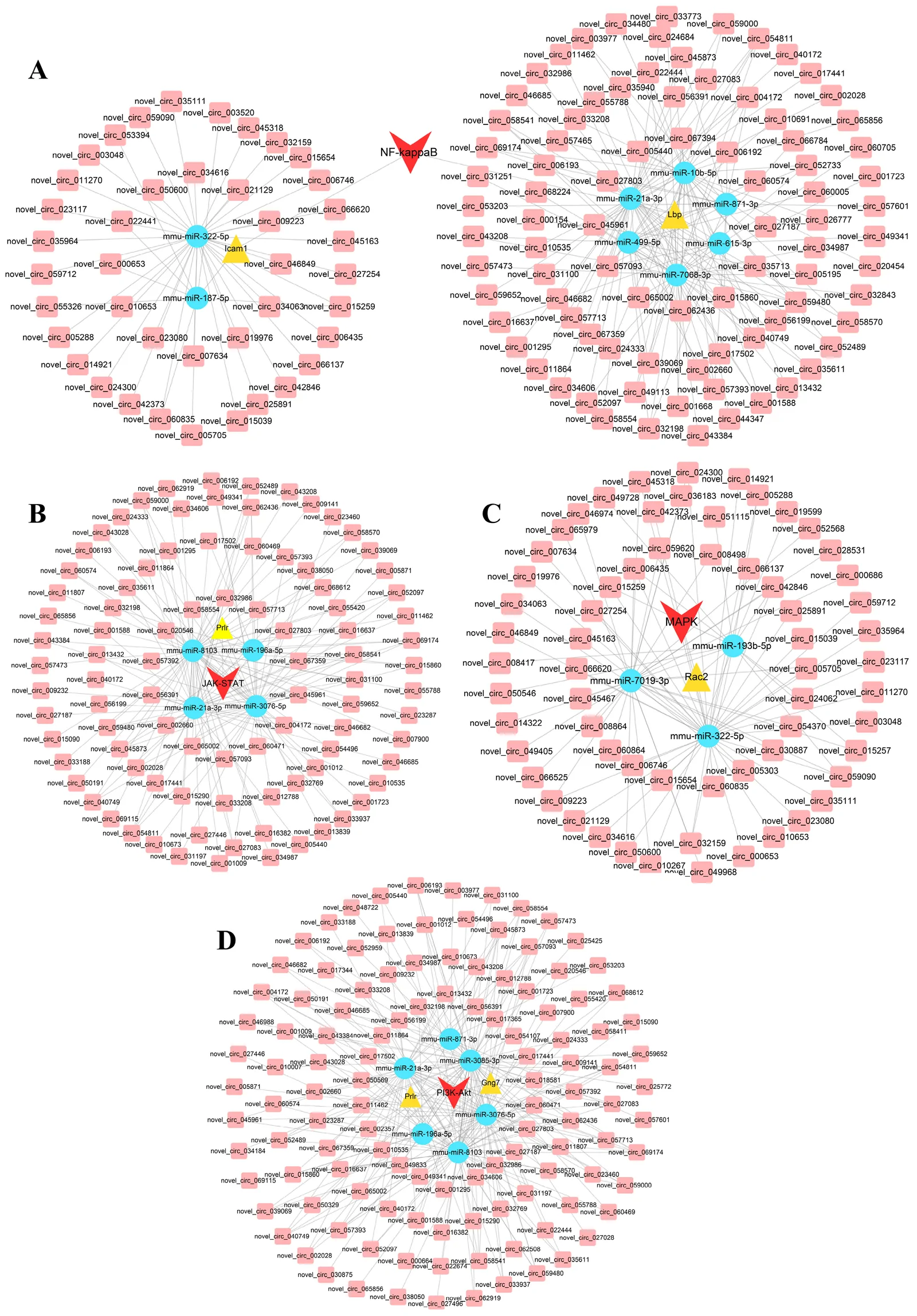
The interaction networks of molecular signatures involved in the inflammatory pathways in *G. parasuis*-infected mouse brain. The networks were constructed based on the predicted regulatory relationships among DEcircRNAs, DEmiRNAs, and DEmRNAs and visualized using Cytoscape (v3.10.1). Red arrows: inflammatory pathways. Yellow triangles: DEmRNAs. Blue circles: DEmiRNAs. Pink squares: DEcircRNAs. (**A**) The interaction network of molecular signatures involved in the NF-kappa B signaling pathway. (**B**) The interaction network of molecular signatures related to the JAK–STAT signaling pathway. (**C**) The interaction network of molecular signatures associated with the MAPK signaling pathway. (**D**) The interaction network of molecular signatures involved in the PI3K–Akt signaling pathway.

To explore the potential regulatory relationships among DEmRNAs, DEmiRNAs, and DEcircRNAs associated with inflammatory signaling pathways in *G. parasuis*-infected mouse brain, inflammatory signaling pathways, including NF-kappa B, JAK–STAT, MAPK, and PI3K–Akt, were first selected based on KEGG enrichment analysis of the source genes of DEcircRNAs (Figure 4B). Subsequently, DEmRNAs involved in these four pathways and their corresponding regulatory DEmiRNAs and DEcircRNAs identified from the sequencing data were selected to construct interaction networks. Network visualization was performed using Cytoscape (v3.10.1) based on the regulatory relationships among these differentially expressed RNA molecules. As shown in Figure 6A–D, four interaction networks were constructed based on the differential molecular signatures associated with the NF-kappa B, JAK–STAT, MAPK, and PI3K–Akt signaling pathways, involving five DEmRNAs, 14 DEmiRNAs, and 207 DEcircRNAs. The analysis showed that Icam1, Lbp, mmu-miR-332-5p, mmu-miR-187-5p, mmu-miR-10b-5p, mmu-miR-21a-3p, mmu-miR-499-5p, mmu-miR-7068-3p, mmu-miR-615-3p, mmu-miR-871-3p, and the corresponding 127 DEcircRNAs were the main interaction molecules in the NF-kappaB network (Figure 6A). Additionally, Prlr, mmu-miR-8103, mmu-miR-196a-5p, mmu-miR-21a-3p, mmu-miR-3076-5p, and the corresponding 92 DEcircRNAs made up the JAK–STAT network (Figure 6B). Rac2, mmu-miR-193b-5p, mmu-miR-7019-3p, mmu-miR-322-5p, and the corresponding 63 DEcircRNAs constituted the MAPK network (Figure 6C). Similarly, Prlr, Gng7, mmu-miR-871-3p, mmu-miR-21a-3p, mmu-miR-3085-3p, mmu-miR-3076-5p, mmu-miR-196a-5p, mmu-miR-8103, and the corresponding 118 DEcircRNAs were the dominant regulatory molecules in the PI3K–Akt network (Figure 6D). The networks suggested that these DEcircRNAs, DEmiRNAs, and DEmRNAs may interact with each other and be involved in the regulation of inflammatory responses during *G. parasuis* infection via NF-kappa B, JAK–STAT, MAPK, and PI3K–Akt signaling pathways.

### 3.7. The ceRNA (DEcircRNAs–DEmiRNAs–DEmRNAs) Regulatory Network in G. Parasuis-Infected Mouse Brain

**Figure 7:**
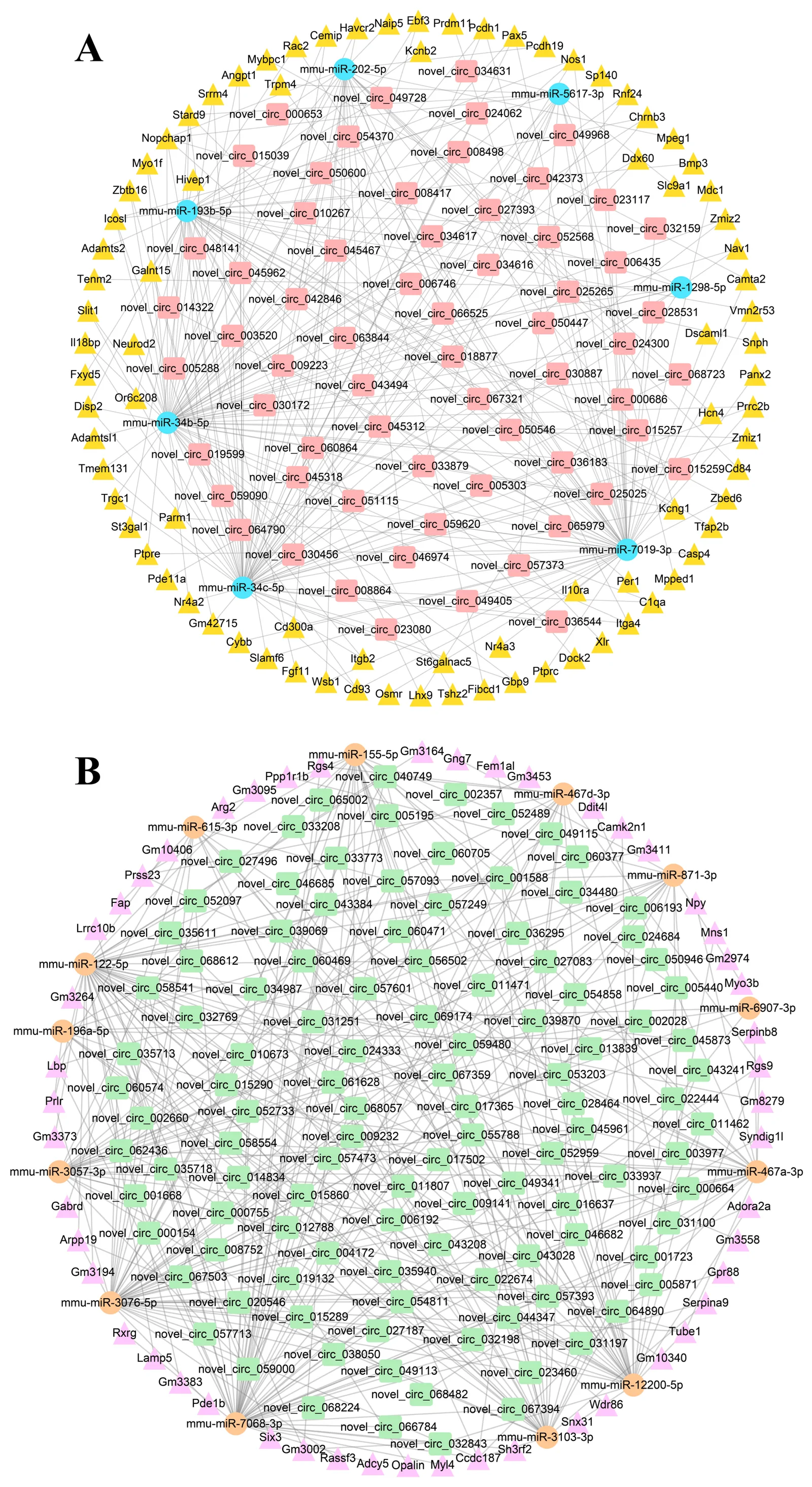
ceRNA regulatory networks (DEcircRNAs–DEmiRNAs–DEmRNAs) in *G. parasuis*-infected mouse brain. Triangles: DEmRNAs. Circles: DEmiRNAs. Squares: DEcircRNAs. (**A**) ceRNA regulatory network composed of elevated circRNAs, decreased miRNAs, and elevated mRNAs in mouse brain following *G. parasuis* infection, including 64 upregulated circRNAs, 7 downregulated miRNAs, 84 upregulated mRNAs, and 268 relationships. (**B**) ceRNA regulatory network of downregulated circRNAs, upregulated miRNAs, and downregulated mRNAs in mouse brain after *G. parasuis* infection, including 113 downregulated circRNAs, 13 upregulated miRNAs, 50 downregulated mRNAs, and 321 relationships.

CircRNAs can function as molecular sponges for miRNAs, reducing their binding to target mRNAs and indirectly elevating the expression of these target mRNAs. According to the sequencing data, we established a ceRNA network focused on the significantly downregulated miRNAs by identifying DEcircRNAs and DEmRNAs that exhibited strong negative correlations (SCC < −0.7). The resulting network comprised 64 upregulated circRNAs, seven downregulated miRNAs, and 84 upregulated mRNAs, interconnected by 268 interactions. Notably, mmu-miR-34c-5p, mmu-miR-34b-5p, mmu-miR-7019-3p, and mmu-miR-193b-5p with a high degree of connectivity emerged as the key regulatory miRNAs within this network (Figure 7A). In addition, another ceRNA regulatory network for the significantly upregulated miRNAs was established by screening for the negatively correlated DEcircRNAs and DEmRNAs (SSC < −0.7), featuring 113 downregulated circRNAs, 13 upregulated miRNAs, 50 downregulated mRNAs, and 321 interactions, with mmu-miR-7068-3p, mmu-miR-3076-5p, mmu-miR-122-5p, and mmu-miR-12200-5p identified as the central regulatory miRNAs (Figure 7B).

### 3.8. The ceRNA (DElncRNAs–DEmirRNAs–DEmRNAs) Regulatory Network in G. Parasuis-Infected Mouse Brain

**Figure 8:**
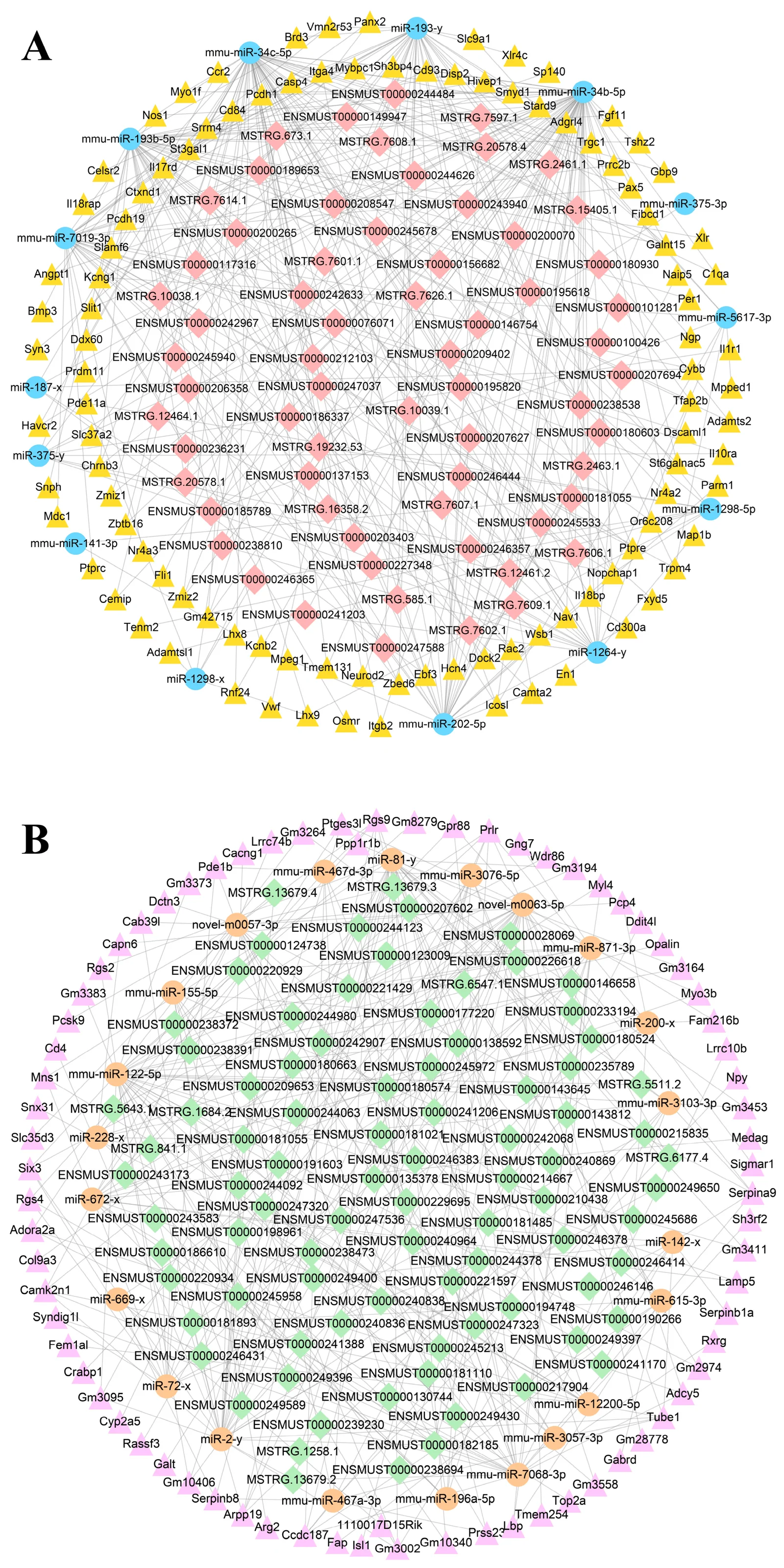
ceRNA regulatory networks (DElncRNAs–DEmiRNAs–DEmRNAs) in the mouse brain after *G. parasuis* infection. Triangles: DEmRNAs. Circles: DEmiRNAs. Diamonds: DElncRNAs. (**A**) ceRNA interaction network composed of increased lncRNAs, decreased miRNAs, and increased mRNAs in mouse brain following *G. parasuis* infection, including 65 upregulated lncRNAs, 14 downregulated miRNAs, 103 upregulated mRNAs, and 318 relationships. (**B**) ceRNA interaction network composed of decreased lncRNAs, increased miRNAs, and decreased mRNAs in mouse brain after *G. parasuis* infection, including 87 downregulated lncRNAs, 22 upregulated miRNAs, 74 downregulated mRNAs, and 299 relationships.

LncRNAs can serve as competitive endogenous RNAs by sequestering miRNAs, thereby alleviating their repressive effects on target mRNAs. Based on this principle, a ceRNA regulatory network was constructed by using the significantly downregulated miRNAs and their negatively correlated DElncRNAs and DEmRNAs (SCC < −0.7). This network consisted of 65 upregulated lncRNAs, 14 downregulated miRNAs, 103 upregulated mRNAs, and 318 interactions. Especially mmu-miR-34b-5p, mmu-miR-34c-5p, mmu-miR-193b-5p, and mmu-miR-7019-3p, with strong connectivity, were identified as the key regulatory miRNAs (Figure 8A). And another ceRNA network for the significantly upregulated miRNAs was established, which consisted of 87 downregulated lncRNAs, 22 upregulated miRNAs, and 74 downregulated mRNAs, with 299 interaction relationships. Notably, mmu-miR-122-5p, mmu-miR-871-3p, mmu-miR-12200-5p, and mmu-miR-7068-3p emerged as the key regulatory miRNAs within this network (Figure 8B).

### 3.9. qRT-PCR Validation of the Differentially Expressed circRNAs, lncRNAs, miRNAs, and mRNAs in Mouse Brain After G. parasuis Infection

**Figure 9:**
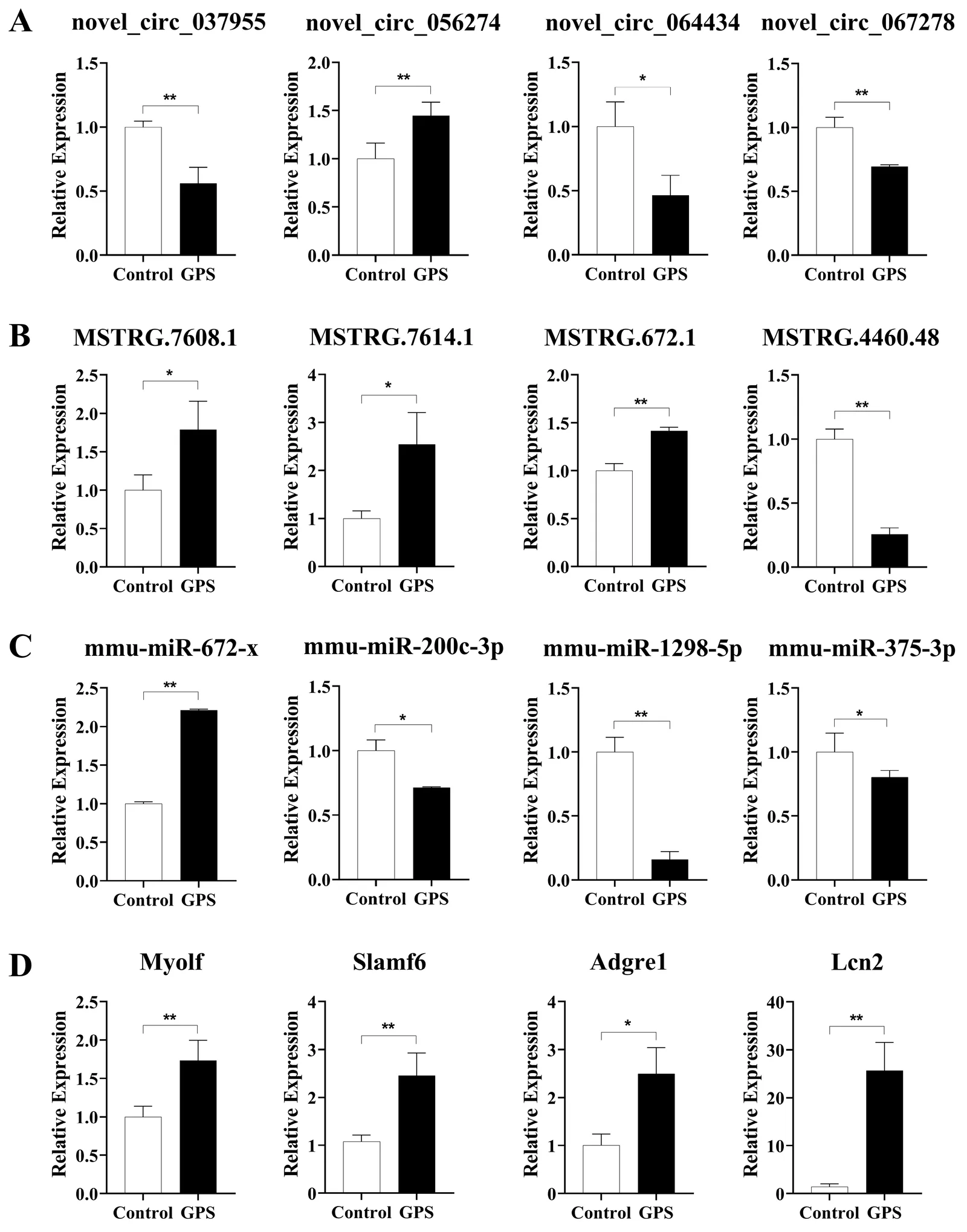
qRT-PCR validation of DEcircRNAs, DElncRNAs, DEmiRNAs, and DEmRNAs in mouse brain after *G. parasuis* infection. (**A**) Relative expression levels of DEcircRNAs (novel_circ_037955, novel_circ_056274, novel_circ_064434, and novel_circ_067278). (**B**) Relative expression levels of DElncRNAs (MSTRG.7608.1, MSTRG.7614.1, MSTRG.672.1, and MSTRG.4460.48). (**C**) Relative expression levels of DEmiRNAs (mmu-miR-672-x, mmu-miR-200c-3p, mmu-miR-1298-5p, and mmu-miR-375-3p). (**D**) Relative expression levels of DEmRNAs (Myolf, Slamf6, Adgre1, and Lcn2). Data are presented as mean ± SD (n = 3). Statistical significance was determined using Student’s *t*-test. * *p* < 0.05 and ** *p* < 0.01 compared with the control group.

**Figure 10:**
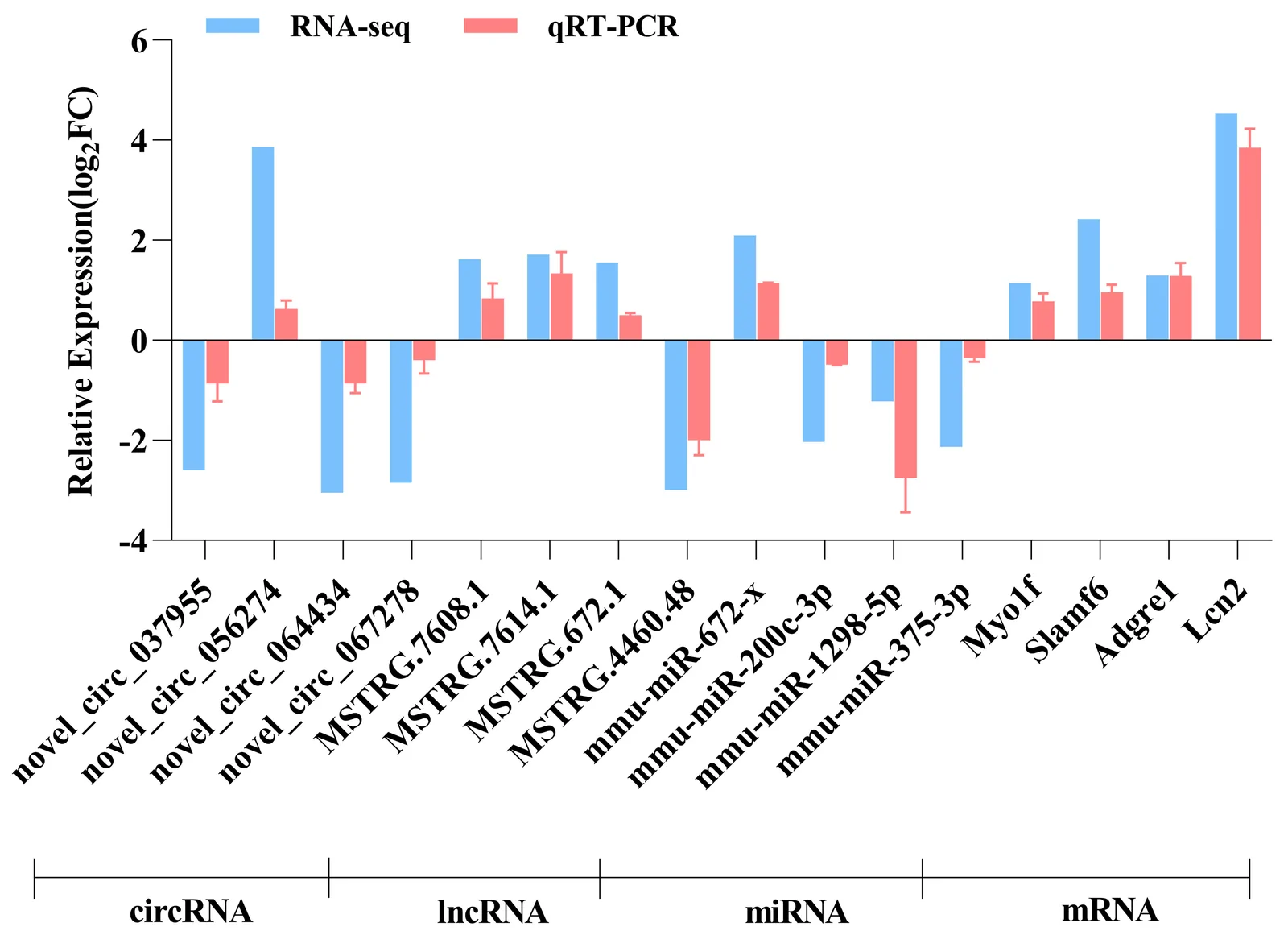
Comparison of the expression levels of circRNAs (novel_circ_037955, novel_circ_056274, novel_circ_064434, and novel_circ_067278), lncRNAs (MSTRG.7608.1, MSTRG.7614.1, MSTRG.672.1, and MSTRG.4460.48), miRNAs (mmu-miR-672-x, mmu-miR-200c-3p, mmu-miR-1298-5p, and mmu-miR-375-3p), and mRNAs (Myolf, Slamf6, Adgre1, and Lcn2) assessed by RNA-seq and qRT-PCR.

To corroborate the results obtained from our transcriptome sequencing, we randomly selected a panel of significantly differentially expressed circRNAs, lncRNAs, miRNAs, and mRNAs for qRT-PCR analysis. The results showed that *G. parasuis* infection significantly downregulated the relative expression of circRNAs, including novel_circ_037955, novel_circ_064434, and novel_circ_067278 (*p* < 0.05), while upregulating the expression level of novel_circ_056274 (*p* < 0.01, Figure 9A). In addition, as shown in Figure 9B, the relative expression levels of lncRNAs including MSTRG.7608.1, MSTRG.7614.1, and MSTRG.672.1 were significantly increased in the *G. parasuis*-infected group (*p* < 0.05), whereas MSTRG.4460.48 expression was significantly decreased (*p* < 0.01). Moreover, compared to the control group, *G. parasuis* infection significantly declined the expression levels of miRNAs, including mmu-miR-200c-3p, mmu-miR-1298-5p, and mmu-miR-375-3p (*p* < 0.05), but significantly enhanced the expression of mmu-miR-672-x (*p* < 0.01) (Figure 9C). Finally, the findings demonstrated that the relative expression levels of mRNAs, including Myolf, Slamf6, and Lcn2, were significantly upregulated in the *G. parasuis*-infected group (*p* < 0.01), and the expression of Adgre1 was also significantly elevated (*p* < 0.05) (Figure 9D). Moreover, the expression trends validated by qRT-PCR closely matched those obtained from sequencing results, confirming the reliability of our sequencing data (Figure 10). 

## 4. Discussion

*G. parasuis* is a highly adaptable pathogen and its ability to disrupt the blood–brain barrier (BBB) and induce meningitis is particularly concerning [28,29]. Increasing evidence has demonstrated that non-coding RNAs (ncRNAs) play crucial roles in regulating disease development and progression [30]. Our study was based on RNA-seq to analyze the impact of *G. parasuis* infection on the ncRNAs (circRNAs, lncRNAs, and miRNAs) expression profiles and the interaction analysis of the ceRNA networks in mouse brain, providing insights into the potential molecular mechanisms underlying *G. parasuis*-induced meningitis. 

Functional enrichment analysis of the source genes of DEcircRNAs was conducted, which was crucial for exploring their functions in biological processes. KEGG analysis indicated that the source genes of DEcircRNAs were mainly enriched in tight junction, neurotrophins, and MAPK signaling pathways. Neurotrophins are a family of proteins crucial for the development, growth, and plasticity of neurons, representing the overseers of brain cell health, with their levels being altered in pathological conditions, such as neuroinflammation [31]. In another study, *G. parasuis* stimulation triggers the transcriptional activation of IL-1α, IL-1β, IL-6, IL-8, and TNF-α through the NF-κB and MAPK pathways in porcine alveolar macrophages (PAMs), thereby triggering inflammation [32]. Thus, we speculated that these key DEcircRNAs and associated signaling pathways might be involved in the inflammatory response elicited by *G. parasuis* infection in mouse brain, although their precise roles and underlying mechanisms require further investigation.

Furthermore, KEGG analysis of the target genes of DEmiRNAs demonstrated that they were primarily engaged in olfactory transduction, phospholipase D, calcium, and mTOR signaling pathways. It has been reported that the absence of phospholipase D2 (PLD2) is involved in the pathogenesis of sepsis-induced cardiomyopathy (SICM) and suppresses myocarditis inflammation and pyroptosis through the NLRP3/caspase 1/GSDMD pathway [33]. Other studies have revealed that, by using astrocytes derived from human induced pluripotent stem cells (hiPSCs), the combination of inflammatory cytokines IL-1α, TNF, and C1q (ITC) induced mTOR activation, resulting in the remodeling of the endolysosomal system and consequently causing the exocytosis of certain endolysosomal cargos [34]. These findings suggested that the DEmiRNAs and associated signaling pathways may be involved in the host response during *G. parasuis* infection; however, the detailed functions require further investigation.

In addition, KEGG analysis illustrated that the target genes of lncRNAs were significantly enriched in cytokine–cytokine receptor interaction, primary immunodeficiency, and the cAMP signaling pathway. It has been observed that in cancer patients, chemotherapy stimulates the production of cytokines through its effects on both normal and tumor cells. The increased production of cytokines damages the integrity of the blood–brain barrier (BBB), thereby allowing circulating cytokines to enter the brain [35], leading to neuroinflammation and neuronal cell death, manifested as cancer-related cognitive impairment (CRCI) [36]. Numerous reports have emphasized the key role of inflammation in the onset and clinical manifestations of primary immunodeficiencies. Diseases with primary immunodeficiency have shown manifestations associated with allergies, inflammation, and autoimmunity, accompanied by lymphocyte proliferation or malignancies [37]. Integrin β1 (ITGB1) has inhibited cartilage inflammation and cell apoptosis through the activation of the cAMP pathway, showing a significant suppressive effect on the progression of osteoarthritis (OA) [38]. Thus, we inferred that these lncRNAs and associated signaling pathways may contribute to the host response during *G. parasuis*-induced meningitis, although their specific functions remain to be elucidated.

CircRNAs possess distinct structural features that allow them to participate in various biogenic processes [39]. This study focused on inflammatory pathways (NF-κB, JAK–STAT, MAPK, and PI3K–Akt signaling pathways) enriched in the source genes of DEcircRNAs in *G. parasuis*-infected mouse brain, and established interaction networks of associated DEmRNAs, DEmiRNAs, and DEcircRNAs (molecular signatures). As transcription factors, the NF-κB family has been reported to regulate the expression of numerous inflammation-related genes critical to kidney disease through binding with the κB elements in promoters and enhances [40]. Additionally, AST has been proven to have anti-apoptotic effects through the activation of the NF-κB signaling pathway, inhibiting microvascular injury through VEGF production, and regulating the MAPK and PI3K–Akt signaling pathways [41]. Moreover, the interaction networks between these inflammation-related signaling pathways and molecular signatures also highlighted Icam1, Lbp, and Gng7 as potential hub molecules within these networks. Icam1 acts as a key mediator in modulating cellular responses during inflammation, injury recovery, and tumorigenesis [42]. Lbp is mainly produced in hepatocytes, acts as a secretory class I acute-phase protein, and plays a crucial role in the innate immune response [43]. In addition, GNG7 has been identified as a negative regulator of tumor growth in lung adenocarcinoma (LUAD) cells via the inhibition of E2F transcription factor 1 (E2F1) [44]. Accordingly, we speculated that these hub genes and associated signaling pathways may be associated with inflammatory responses during *G. parasuis* infection.

Increasing evidence indicates that ncRNAs including circRNAs, lncRNAs, and miRNAs are pivotal in regulating the progression of disease [30]. Therefore, we constructed the ceRNA (DEcircRNAs/DElncRNAs–DEmiRNAs–DEmRNAs) in mouse brain after *G. parasuis* infection based on transcriptome sequencing results. The DEcircRNAs/DElncRNAs–DEmiRNAs–DEmRNAs interaction networks suggested potential regulatory relationships among circRNAs, lncRNAs, miRNAs, and mRNAs. The key regulatory DEmiRNAs, such as mmu-miR-34b-5p, miR-122-5p, and mmu-miR-193b-5p, emerged as potential hub miRNAs within these networks. Studies have shown that miR-34b-5p participates in inflammatory pathways such as the IL-17 signaling pathway, suggesting that it could be an important factor in the progression of idiopathic pulmonary arterial hypertension (IPAH) by promoting inflammation [45]. Inhibition of miR-122 has been reported to reduce lipid accumulation and inflammation in Hepatocyte HL-7702 (L02) cells induced by oleic acid (OA) by suppressing the TLR4/MyD88/NF-κBp65 signaling pathway [46]. Additionally, inhibition of miR-193b-5p in primary human pulmonary microvascular endothelial cells prevented TNF-induced downregulation of occludin gene and protein expression, as well as the loss of barrier function [47]. These studies provide further support for our hypothesis that the DEcircRNAs/DElncRNAs–DEmiRNAs–DEmRNAs networks may be involved in the regulation of inflammatory responses in mouse brain following *G. parasuis* infection. 

Our findings indicated that ncRNAs (circRNAs, lncRNAs, and miRNAs) are involved in the host response during *G. parasuis*-induced meningitis in mouse brain, and that *G. parasuis* infection leads to changes in ncRNA expression profiles. Furthermore, the identified ceRNA (DEcircRNAs/DElncRNAs–DEmiRNAs–DEmRNAs) interaction networks may contribute to the modulation of inflammation in mouse brain following *G. parasuis* infection. 

Several limitations of the present study should be acknowledged. A formal a priori statistical power analysis was not performed. In addition, the histopathological assessment was based on qualitative morphological observations, and animal-level semiquantitative scoring could not be retrospectively performed. Thus, the observed inflammatory cell accumulation should be interpreted with caution. Further functional studies are required to validate the specific roles of these ncRNAs and clarify the underlying regulatory mechanisms during *G. parasuis*-induced meningitis. Moreover, transcriptome sequencing was performed at a single time point (72 h post-infection), and dynamic changes in RNA expression during different stages of infection remain to be investigated. Only female mice were used in the present study to maintain consistency with our previously established model. Therefore, potential sex-related differences in immune, inflammatory, and transcriptomic responses could not be evaluated, and the findings may not be fully generalizable to male mice. Finally, as the present study used a mouse model to investigate host neuroinflammatory responses, further validation in the natural porcine host will be valuable to better understand the relevance of these findings to *G. parasuis* infection in pigs.

## 5. Conclusions

This study systematically analyzed the expression changes in circRNAs, lncRNAs, miRNAs and mRNAs in a *G. parasuis*-induced mouse meningitis model. A series of differentially expressed RNAs and inflammation-related signaling pathways were screened out, and two types of ceRNA regulatory networks (DEcircRNAs/DElncRNAs–DEmiRNAs–DEmRNAs) were successfully constructed. qRT-PCR verified the reliability of transcriptome data. Our results provide insights into the potential involvement of ncRNA-mediated ceRNA networks in *G. parasuis*-induced neuroinflammation and provide potential candidates for further investigation of the molecular mechanisms underlying *G. parasuis*-induced meningitis.

## Figures and Tables

**Table 1:** Primer sequences used for qRT-PCR analysis of differentially expressed genes (DEGs).

Gene		Primer Sequences (5′ → 3′)
novel_circ_037955	Forward	TAATGAAGTGAGCCAGTTTGC
	Reverse	GATGTCCTCGGCTGTTTCC
novel_circ_056274	Forward	ATCCTTCTTTGCCTTTATCAGC
	Reverse	AAATAACACCTGCCTGTCTCAC
novel_circ_064434	Forward	CAGTGTTGTGACGGATGGTG
	Reverse	CTTGGAGGTTGTTGTCTGTGAT
novel_circ_067278	Forward	CGCCACCAAAGTTAATCAGA
	Reverse	TGAGCACATAGGGAGCATTT
MSTRG.7608.1	Forward	CTCAGCAGATTGTCAGTCCC
	Reverse	CATCAGTAGTCCTGGCGTCC
MSTRG.7614.1	Forward	AAGAGATGACCAGTGAAGACCAGAC
	Reverse	GCTGAAGACAGACGAGTGAGGAG
MSTRG.672.1	Forward	CTACACGACAGTGAGGGACG
	Reverse	CGATAGCAGGTTGGGATGA
MSTRG.4460.48	Forward	TCGGTCGGTCGGTAGTCCTTC
	Reverse	CCAGCAGGCTAGTCTCCATTCAC
mmu-miRNA-672-x	Forward	GCCGAGTTTGTTCGTTCGGCTC
	Reverse	CTCAACTGGTGTCGTGGAGTC
mmu-miRNA-200c-3p	Forward	AAACAAACCAGAGGCTCACACT
	Reverse	CTCAACTGGTGTCGTGGAGTC
mmu-miRNA-1298-5p	Forward	TTTGTTCGTTCGGCTCGCGTGA
	Reverse	CTCAACTGGTGTCGTGGAGTC
mmu-miRNA-375-3p	Forward	TTCATTCGGCTGTCCAGATGTA
	Reverse	CTCAACTGGTGTCGTGGAGTC
Myolf	Forward	TTTCACTGGCAGAGTCACAAC
	Reverse	TGCTTGAAGGGGTTTACAGA
Slamf6	Forward	GCAAATCAGCAACCTTACCA
	Reverse	CAGTCGTTCAAAGACCCTCA
Adgre1	Forward	ACAACTGAATGGGTGGTGAG
	Reverse	TGGTGTTAAGACAGGTGGATG
Lcn2	Forward	CAGTCAGATGATTCAGACGGAG
	Reverse	CAGTGCCACCAACAACACG
GAPDH	Forward	AAGCCCATCACCATCTTCCA
	Reverse	CACCAGTAGACTCCACGACA
U6	Forward	GGAACGATACAGAGAAGATTAGC
	Reverse	TGGAACGCTTCACGAATTTGCG

## Data Availability

The original contributions presented in this study are included in the article. Further inquiries can be directed to the corresponding authors.
